# Pilot study of Lokomat versus manual-assisted treadmill training for locomotor recovery post-stroke

**DOI:** 10.1186/1743-0003-6-18

**Published:** 2009-06-12

**Authors:** Kelly P Westlake, Carolynn Patten

**Affiliations:** 1 Department of Radiology and Biomedical Imaging, University of California, San Francisco, California, USA; 2 Brain Rehabilitation Research Center, Malcolm Randall VA Medical Center, Gainesville, Florida, USA; 3 Department of Physical Therapy, University of Florida, Gainesville, Florida, USA

## Abstract

**Background:**

While manually-assisted body-weight supported treadmill training (BWSTT) has revealed improved locomotor function in persons with post-stroke hemiparesis, outcomes are inconsistent and it is very labor intensive. Thus an alternate treatment approach is desirable. Objectives of this pilot study were to: 1) compare the efficacy of body-weight supported treadmill training (BWSTT) combined with the Lokomat robotic gait orthosis versus manually-assisted BWSTT for locomotor training post-stroke, and 2) assess effects of fast versus slow treadmill training speed.

**Methods:**

Sixteen volunteers with chronic hemiparetic gait (0.62 ± 0.30 m/s) post-stroke were randomly allocated to Lokomat (n = 8) or manual-BWSTT (n = 8) 3×/wk for 4 weeks. Groups were also stratified by fast (mean 0.92 ± 0.15 m/s) or slow (0.58 ± 0.12 m/s) training speeds. The primary outcomes were self-selected overground walking speed and paretic step length ratio. Secondary outcomes included: fast overground walking speed, 6-minute walk test, and a battery of clinical measures.

**Results:**

No significant differences in primary outcomes were revealed between Lokomat and manual groups as a result of training. However, within the Lokomat group, self-selected walk speed, paretic step length ratio, and four of the six secondary measures improved (*p *= 0.04–0.05, effect sizes = 0.19–0.60). Within the manual group, only balance scores improved (*p *= 0.02, effect size = 0.57). Group differences between fast and slow training groups were not revealed (*p *≥ 0.28).

**Conclusion:**

Results suggest that Lokomat training may have advantages over manual-BWSTT following a modest intervention dose in chronic hemiparetic persons and further, that our training speeds produce similar gait improvements. Suggestions for a larger randomized controlled trial with optimal study parameters are provided.

## Background

Stroke is the leading cause of serious, chronic disability in the United States and Canada. While two-thirds of people who suffer a stroke regain ambulatory function, the resulting gait pattern is typically asymmetrical, slow, and metabolically inefficient [1,2]. These characteristics are associated with difficulty advancing and bearing weight through the more affected limb, leading to instability and an increased risk of falls [3]. Secondary impairments, including muscle disuse and reduced cardiorespiratory capacity, often contribute to further functional declines in gait. Hence, improved walking is one of the most frequently articulated goals of rehabilitation and interventions that effectively enhance locomotor function are essential to improve quality of life for many stroke survivors and their families [4,5]. Nevertheless, the effectiveness of locomotor training still remains unclear and the need to conduct randomized controlled trials to definitively answer this question is paramount. To best determine the key parameters of such a large-scale study, preliminary data must first be collected in the form of a pilot study.

Manually-assisted body-weight supported treadmill training (BWSTT) is a contemporary approach to gait rehabilitation wherein an individual walks on a treadmill with body-weight partially supported by an overhead harness. One to three therapists/trainers manually facilitate hemiparetic limb and trunk control in an effort to normalize upright, reciprocal stepping and dynamic postural control. Advantages of this approach are that little to no ambulatory function is required to initiate locomotion and early post-stroke training effects are transferred to improvements in overground gait including: symmetry, speed, and endurance as well as motor impairment and balance scores [6,7]. These positive outcomes can be maintained even at 6 months post-locomotor training [8]. However, because locomotor training involves repetition of hundreds of steps within one session, facilitation of a symmetrical, patterned gait can be very labor intensive for both therapist(s) and participant and further, presents a non-trivial risk of injury to the trainers. Moreover, the repetition of kinematically consistent stepping patterns is hindered by inconsistencies in motor performance of the therapists assisting movement. Conflicting evidence within 15 randomized controlled trials comparing BWSTT and traditional gait training (i.e. overground gait training, motor relearning) in persons post-stroke highlight the difficulty in interpreting the effectiveness of manually applied cues during repetitive stepping [9].

In response to the challenges presented in administering manual-BWSTT, robotic devices, such as the Lokomat^® ^(Hocoma, Inc., Zurich, Switzerland), have recently emerged as a means to automate locomotor training in neurorehabilitation. Using robotic assistance, an exoskeleton facilitates a bilaterally symmetrical gait pattern as the individual actively attempts to advance each limb while walking on the treadmill. The preprogrammed walking pattern corresponds with normal gait kinematics including: gait cycle timing (i.e. stance vs. swing phase), inter-limb and inter-joint coordination, appropriate limb loading, and afferent signaling [10]. Animal models have demonstrated that afferent signals derived from limb movement and loading converge at the level of the spinal cord to trigger and control locomotor pattern generators (LPGs) [11,12]. Previous work in persons with spinal cord injuries underscores the importance of the accuracy of relevant timed peripheral inputs to induce changes in locomotor function [13]. Accordingly, the rhythmic and repetitive stepping pattern provided by robotic assistance, combined with active limb loading and kinematic consistency has been shown to promote plasticity of LPGs at the spinal cord level [14] as well as supraspinal structures [15]. Still, despite recent interest in automated locomotor training, there remains very little evidence to support the superiority of this technique over traditional gait training.

Previous comparisons between robotic-BWSTT and manual-BWSTT, overground gait training, and traditional approaches result in equivocal findings based, in part, on differences in outcome measures, subject characteristics, and gait training protocols [16-20]. However, separation of the general effects of locomotor training from true automated training effects requires standardization of BWSTT parameters, i.e. BWS percentage and stiffness, treadmill speed, and use of handrails [21], and a comparison between the application of manual or robotic limb guidance with the intent of approximating normal gait kinematics in a well-defined subject population. In controlling these variables, we hypothesize that Lokomat training will produce greater improvements in gait speed and symmetry than manual training.

Extending the notion of task-specificity underlying both Lokomat and manual-BWSTT, one particular variable of interest is training speed. If the therapeutic goal is increased overground walking speed, then training must occur at speeds that exceed habitual overground walking speed for a person with hemiparesis. The majority of the current models of the Lokomat robotic orthosis offer treadmill belt speeds up to 0.83 m/s (3 km/h), thus it is yet unknown whether training at higher Lokomat speeds produces similar positive gait changes as revealed in earlier studies [8,22]. Here, we hypothesize that the addition of external timing cues and kinesthetic input induced by Lokomat training and manual BWSTT at training speeds of up to 1.4 m/s (5 km/h) will produce greater improvements in spatio-temporal gait parameters, postural control, and clinical outcomes than groups trained at slower speeds.

Objectives of this pilot study were to: 1) compare the efficacy of Lokomat versus manual assisted-BWSTT in persons with chronic locomotor deficits post-stroke and 2) probe the effect of locomotor training at speeds corresponding with overground gait in non-disabled individuals to habitual self-selected walking speed in persons post-stroke. Since self-selected overground gait speed and step length symmetry are important indicators of locomotor performance, and further, are related to function and quality of life following stroke [23,24], these variables were selected as our primary variables of interest. Secondary outcomes included fast overground walking speed, a battery of clinical and functional measures, and a quality of life indicator.

## Methods

### Participants

Sixteen persons with hemiparesis resulting from a single cortical or subcortical stroke (confirmed by CT or MRI) greater than 6 months prior to the study, who were categorized as at least unlimited household ambulators (e.g. > 0.3 m/s) [4] participated. Exclusion criteria included: 1) unstable cardiovascular, orthopedic, or neurological conditions, 2) uncontrolled diabetes that would preclude exercise of moderate intensity, or 3) significant cognitive impairment affecting the ability to follow directions. Participants were recruited from local hospitals, rehabilitation centers, and stroke associations. All procedures were approved by the Stanford University Institutional Review Board and all participants provided written, informed consent prior to study involvement.

### Allocation Procedures

In an effort to achieve our primary research goal, participants were randomized into either a Lokomat (n = 8) or manual (n = 8) group using a computer-generated random order. To reach our secondary goal, an equal number of participants within each group were randomly assigned to either a fast (n = 8) or slow (n = 8) training group. The randomization list was overseen by one of the investigators (CP) who had no contact with participants until group assignment was revealed. Further, group assignment was not revealed to study personnel until the participant was consented and baseline testing was complete.

### Intervention

Both groups received 12 sessions (3×/wk over 4 weeks) involving 30 min of stepping per session. At least one 2–3 minute break was provided after 15 min. Total set-up and treatment time never exceeded 1 hr. Training speeds were maintained below 0.69 m/s (2.5 km/h) in the slow groups and above 0.83 m/s (3 km/h) in the fast groups. Within the fast groups, locomotor training was either started at 0.83 m/s or progressed to this speed as early as possible (e.g. by Session 3) while maintaining gait quality, i.e. symmetrical, foot clearance, without knee buckling. Treadmill speed was progressed in 0.2 km/hr increments approximately every 5 min as long as the above-mentioned gait quality was observed by the therapists. If a new high speed could not be maintained for an extended period, training would ensue in 2–3 minute intervals at the higher speed followed by 2–3 minutes at a lower speed. BWS was initiated at 35%. The Lokomat system used for this study includes the Lokolift, a compliant, electromechanical body-weight support system that monitors and adjusts unweighting in real time to maintain BWS at the prescribed level. This BWS system contrasts with the stiff, counterweighted support system used in the original Lokomat models. A compliant system adjusts to the participant's center of gravity throughout the gait cycle, enabling vertical pelvic movement similar to overground gait, supporting symmetrical movement and producing kinetics similar to overground walking [21,25]. If the maximal treadmill speed, 0.69 m/s (2.5 km/h) in the slow group or 1.4 m/s (5 km/h) in the fast group, was reached, BWS was reduced in increments of 5% as long as gait quality was maintained. Our goal during training was to improve gait kinematics. To achieve this objective, all participants trained without an ankle-foot orthosis, assistance was reduced once safety was no longer a concern, and rest periods were provided if gait quality was noted to deteriorate. In addition, handrail use has been shown to significantly alter the gait pattern and thus was strongly discouraged [25].

Participants assigned to the Lokomat group trained in a robotic orthosis. Thigh and leg straps secured the Lokomat exoskeleton to the participant; motors on each robotic leg facilitated movement of the hip and knee joints with trajectories programmed by the manufacturer based on a single, healthy individual's gait pattern. Only when necessary to maintain foot clearance, the ankle was maintained in neutral dorsiflexion by means of an elastic foot strap. Force sensors within the Lokomat hip and knee joints provided output on a visual display that was monitored by the treating physical therapist. In an effort to maintain consistency in training parameters, Lokomat assistance was provided at 100% bilateral guidance force for all participants throughout all training sessions. Participants were provided verbal encouragement to actively step in conjunction with the movement presented by the Lokomat.

Participants in the manual-BWSTT group were treated by 1–2 skilled physical therapists/trainers who provided manual guidance of the more affected limb, trunk stabilization/alignment, and verbal and visual cues to normalize stepping kinematics. Our intent in using this number of therapists was to mimic clinic feasibility and training in previous reports [20]. The target gait pattern included: adequate trunk alignment, weight shift, acceptance to and from the paretic limb and temporal symmetry between limbs. The treating therapist individualized treatment to facilitate trunk and limb control throughout the gait cycle. Common cues included coaching to: increase plantarflexion propulsion and/or hip flexion at swing initiation, increase dorsiflexion and knee extension at heel strike, and maintain neutral knee alignment (i.e. avoid hyperextension) at midstance. A second trainer provided pelvic stabilization and assistance with weight shift/acceptance as needed. Participants in both groups were provided visual feedback via a full-length mirror placed at the front of the treadmill.

### Measurement

Participants were assessed before and after the 4-week intervention. Self-selected overground walking speed and fast overground walking speed were recorded using a 4.3 m GaitRite mat (CIR Systems, Havertown, Pennsylvania, USA). Participants walked an additional 0.5 m on both ends of the walkway to allow for acceleration and deceleration and were instructed to walk either: "as if taking a comfortable walk in the park" for self-selected walking speed or "as if they were in the middle of the intersection and the light had just changed to red" for fast walking speed. The mean of 3 trials was calculated. Step length of the paretic (P) and nonparetic (NP) limb was also recorded and later used to calculate absolute (ABS) step length asymmetry during self-selected walking speed as follows:



This calculation is a modification of the paretic step length ratio (SLR) [26] and can range from 0 to 1, with an index of 0 reflecting perfect symmetry. The 6-minute walk test was recorded as a measure of gait endurance. Participants were instructed to cover as much distance as possible within a 6-minute period while walking safely. This test was completed along a level carpeted corridor with one turn-around point every 39 meters. For all overground gait assessments, ambulation without an assistive device or lower extremity orthoses was encouraged. However, use of these assistive devices was allowed if deemed necessary for safety. Device usage was consistent between pre- and post-testing.

Secondary outcomes were selected to target impairment, activity, and participation according to the World Health Organization classifications. Motor impairment was evaluated with the lower extremity Fugl-Meyer assessment, which is a valid and reliable measure in persons post-stroke [27,28]. Activities were assessed with the short physical performance battery and the Berg Balance Scale. The short physical performance battery produces a summary score (range 0–12) reflecting scores on 3 timed tasks: walking 8-ft, rising from a chair 5 times, and maintaining a static posture (feet together, semi-tandem, tandem) [29]. Good to excellent reliability and predictive validity have been demonstrated for these tests [30,31]. The Berg Balance Scale is comprised of 14 static and dynamic balance tasks with a maximum score of 56. This measure demonstrates good reliability and validity in a population post-stroke [32,33]. Participation in life events was assessed using the Late Life Function and Disability Instrument (LLFDI) [34], which is composed of a disability section assessing limitation and frequency of performance and a function section measuring difficulty in performing certain physical tasks. Good reliability and validity has also been demonstrated for this measure in a population with a range of functional limitations [35,36].

### Data Analysis

Statistical analyses were conducted using SPSSv15.0 (SPSS, Inc., Chicago, Illinois, USA). Given the small sample size, non-parametric statistics were used. Baseline characteristics between groups were compared using the Mann-Whitney U test for continuous and ordinal variables and the Fisher's exact test for categorical variables. Between group comparisons (Lokomat vs. Manual groups and Fast vs. Slow Training groups) were assessed with the Mann-Whitney U-test using pre-post change scores in ordinal variables. Within group comparisons (Pre vs. Post training) were assessed using the Wilcoxon Signed Ranks Test. Statistical significance was established at α < 0.05.

To determine whether a statistically significant difference is of practical concern, effect sizes and percent change were calculated. Effect sizes were calculated as the difference between the means of the two groups (Lokomat and manual) or between the mean pre-test and post-test values of the same group divided by the common standard deviation (SD) at pre-test. Results were interpreted following standards established by Cohen [37] where 0.2 is indicative of a small effect, 0.5 a medium, and 0.8 a large effect size.

## Results

All sixteen participants completed the study and the twelve training sessions were well tolerated with two exceptions. First, following the eleventh session, one participant in the manual group complained of ankle pain on the hemiparetic side and failed to complete the final training session. Second, despite using a regular rotation of two treating therapists, one therapist suffered a repetitive strain injury of the rotator cuff while training the third manual group participant. Participant characteristics are enumerated in Table 1. Group equivalency (i.e. Lokomat vs. manual and fast vs. slow) was established with no significant baseline differences, *p *≥ 0.13. In the Lokomat group removal of the foot strap was possible in 3 participants. One participant advanced to walking without the foot strap for approximately 54% of sessions, while two additional participants advanced to no foot strap for 25% of sessions.

**Table 1:** Baseline characteristics and training parameters

	**Lokomat (n = 8)**	**Manual (n = 8)**	***p *value**
Age, mean (SD), y	58.6 (16.9)	55.1 (13.6)	0.72^a^
Women, n (%)	2 (25)	1 (12.5)	1.0^b^
Time since stroke, mean (SD), mo	43.8 (26.8)	36.8 (20.3)	0.72^a^
Stroke location, n			
MCA territory (multiple locations)	3	4	1.0^b^
Frontal lobe	0	1	1.0^b^
Temporoparietal lobe	1	0	1.0^b^
Parietal lobe	1	1	1.0^b^
Basal Ganglia	3	1	1.0^b^
Thalamus	0	1	1.0^b^
Pons	0	1	1.0^b^
Type of Stroke, n			
Ischemic	3	5	1.0^b^
Hemorrhagic	5	3	1.0^b^
Left sided hemiparesis, n	4	5	1.0^b^
LE Fugl-Meyer total score, mean (SD)	83.3 (7.3)	80.6 (6.3)	0.13^a^
Self-selected walking speed, mean (SD), m/s	0.62(0.31)	0.62 (0.28)	0.80^a^
Training speed, mean (SD), m/s	0.82 (0.2)	0.67 (0.2)	0.16^a^
Training BWS, mean (SD)	29.4 (5.9)	31 (9.1)	0.28^a^

^a ^Mann-Whitney U test;^b ^Fisher's exact test

Our first aim of this pilot study was to compare the effectiveness of Lokomat versus manual-assisted BWSTT on gait-related outcomes. Overall results revealed no significant differences between Lokomat and manual training group improvements on self selected walk speed, *p *= 0.72, absolute paretic step length ratio, *p *= 0.28, or secondary variables, *p *= 0.54–0.96. However, within the Lokomat group, a greater number of variables demonstrated significant pre- vs. post-test differences compared with the manual group (Table 2).

**Table 2:** Group Comparisons of selected outcomes

**Variable**	**Lokomat Group (n = 8)**		**Manual Group (n = 8)**	
	
	**Pre-Test**	**Post-Test**	**Pre-Test**	**Post-Test**
**SSWS (m/s)**	0.62 ± 0.31 (0.26–1.04)	0.72 ± 0.38* (0.3–1.38)	0.62 ± 0.28 (0.24–0.91)	0.65 ± 0.29 (0.30–1.02)
**FWS (m/s)**	0.87 ± 0.55 (0.32–1.85)	0.96 ± 0.66* (0.33–2.28)	0.72 ± 0.37 (0.26 ± 1.2)	0.70 ± 0.33 (0.35–1.13)
**6 MWT (m)**	267.3 ± 187.2 (71–625.5)	278.1 ± 176.5 (89.3–638.0)	234.3 ± 141.2 (66.4–452.7)	212.4 ± 113.5 (86.5–362.9)
**SLR_abs_**	0.53 ± 0.58 (0.03–1.87)	0.37 ± 0.46 * (0.06–1.46)	0.39 ± 0.37 (0.05–1.10)	0.34 ± 0.35 (0.02–1.04)
**LE FM (/35)**	23.0 ± 4.3 (15–28)	25.6 ± 5.0 * (19–34)	21.4 ± 5.1 (14–29)	22.4 ± 5.2 (14–29)
**SPPB (/12)**	6.9 ± 3.4 (2–12)	7.9 ± 3.2 * (4–12)	7.8 ± 3.0 (4–12)	8.5 ± 3.1 (4–12)
**BBS (/56)**	46.9 ± 7.5 (38–56)	48.3 ± 6.8 * (41–56)	47.0–7.0 (38–55)	51.0–5.4 † (40–56)
**LLFDI**				
**Disability**				
***Frequency*** **(/100)**	52.6 ± 8.1 (41.4–65.1)	52.8 ± 7.9 (41.4–62.3)	49.7 ± 11.4 (29.2–70.6)	53.5 ± 10.2 (35.2–66.7)
***Limitation*** **(/100)**	59.4 ± 7.0 (49.2–69.2)	62.3 ± 8.5 (49.9–71.3)	61.6 ± 9.1 (46.4–75.6)	66.4 ± 10.2 (47.9–77.6)
**Function**	51.3 ± 7.7	52.0 ± 8.6	48.6 ± 6.0	54.1 ± 4.8
**(/100)**	(43.1–64.0)	(42.5–66.8)	(41.9–58.7)	(46.1–61.6)

Note: values are mean ± SD (range)

* Pre-post difference within Lokomat group, *p *< 0.05; † difference within manual group, *p *< 0.05

Abbreviations: SSWS = self selected walking speed; FWS = Fast walking speed; 6 MWT = 6 minute walk test; SLR_abs _= Absolute step length ratio; LE FM = Lower extremity Fugl-Meyer; SPPB, short physical performance battery; BBS = Berg Balance Scale; LLFDI = late life function and disability instrument.

**Figure 1:**
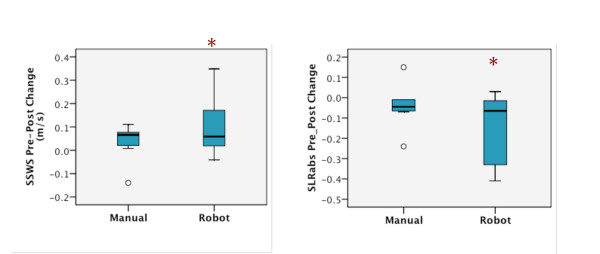
**Medians and lower and upper quartiles for pre-post differences in the manual and Lokomat group**. A. Self-selected walk speed. B. Absolute step length ratio (negative change scores represent a shift towards symmetrical step lengths). Extreme values are greater than 3 times the interquartile distance.* Significant difference only within the Lokomat group (p < 0.05).

Although an equal number of participants (n = 7) produced a training related increase in self-selected overground walking speed in each group, a significant difference, *p *= 0.04, was revealed only in the Lokomat group with a pre-post intervention difference of 0.10 m/s and an effect size of 0.32 which contrasted with a 0.03 m/s difference and 0.11 effect size in the manual group (Figure 1A; Table 2).

Absolute paretic step length ratio (SLR_abs_) during self-selected overground walking speed was also significantly reduced (i.e. closer to an SLRabs of 0) from pre- to post-test in the Lokomat group, *p *= 0.05, effect size 0.26, reflecting improved symmetry in 6 of 8 Lokomat group participants (Figure 1B; Table 2).

**Figure 2:**
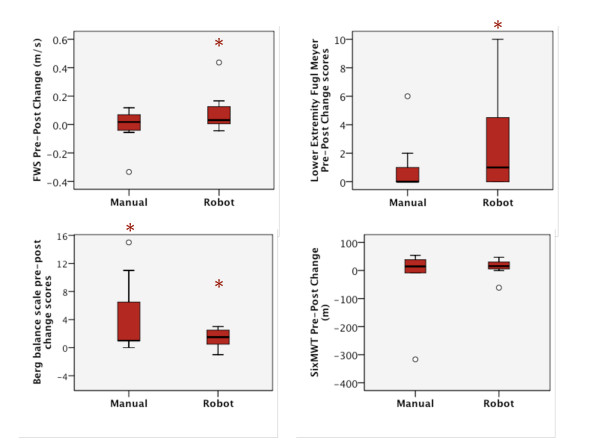
**Medians and lower and upper quartiles for pre-post differences in the manual and Lokomat group**. A. Fast Walk speed. B. Lower Extremity Fugl-Meyer scores (higher scores represent improved sensorimotor recovery). C. Berg Balance Scale (higher scores represent improved balance). D. Six minute walk test (distance covered). * Significant difference within Lokomat group between pre- and post-test (p < 0.05). † Significant difference within manual group between pre- and post-test (p < 0.05).

With the exception of the 6-minute walk test and LLFDI, *p *≥ 0.16, all secondary measures revealed significant improvements within the Lokomat group, yet only one improvement was noted in the manual group (Table 2). Fast overground walk speed improved from pre- to post-training in 6 of 8 Lokomat participants, *p *= 0.05, with a small effect size of 0.15 (Figure 2A). Lower extremity Fugl-Meyer score improvements were also noted, *p *= 0.04, with an effect size of 0.60 and higher scores in 5 of 8 Lokomat group participants (Figure 2B). Similarly, short physical performance battery scores were improved in the Lokomat group, *p *= 0.04, with an effect size of 0.29 and improvements in 5 of 8 participants. Berg Balance Scale scores significantly improved in both the Lokomat group, *p *= 0.04, effect size 0.19 (5 participants improved), and manual group, *p *= 0.02, effect size 0.57 (7 participants improved) (Figure 2C).

Participation in life events, as measured by the LLFDI, also demonstrated improvements, but statistical differences were attained only as a main effect of visit (i.e. pre vs. post) and were not specific to either the Lokomat or manual-BWSTT group. The disability component reflects two dimensions: limitation and frequency. Our data revealed that participants felt less limited in their participation in activities at home and in the community, *p *= 0.02, with an effect size of 0.49. Interestingly, the frequency of self-reported participation in these tasks remained unchanged from pre-test to post-test, *p *≥ 0.11. Participants also perceived less difficulty in terms of the performance of certain functional tasks including: dressing, walking a mile, and climbing stairs, *p *= 0.004, with an effect size of 0.47.

In an effort to differentiate changes in motor control from aerobic conditioning effects, we conducted two post-hoc correlations: 1) self-selected overground walking speed and 6-minute walk test, and 2) Berg Balance Scale and 6-minute walk test. Due to the influence of gait speed and balance in a chronic population (mean 5.5 years post-stroke), previous work has cautioned against interpreting the 6-minute walk test as an indicator of aerobic capacity [38]. However, in our study of participants who averaged 3.3 years post-stroke, no significant relationship was identified between either changes in 6-minute walk test and self selected gait speed, *r *= 0.14, *p *= 0.61, or changes in 6-minute walk test and balance (Berg Balance Scale), *r *= 0.27, *p *= 0.32. Therefore, improvements in gait speed and balance detected in the present study could be attributed to enhanced locomotor control and were not likely due to changes in endurance as measured using the 6-minute walk test.

Our second aim was to assess locomotor-training effects at faster vs. slower treadmill speeds. As anticipated, independent of whether training occurred in the Lokomat or manual-BWSTT mode, average weekly training speeds within the fast and slow groups were similar, *p *≥ 0.29 (Table 3). Therefore, data from both Lokomat and manual groups were collapsed to isolate the effects of training speed. On average, participants in the fast group trained at speeds that were 50% above their baseline overground walking speed, while the training speed in the slow group was similar to their baseline overground walking speed. Despite these differences in absolute and relative between-group training speeds, no group differences were noted on primary or secondary outcome measures, *p *≥ 0.28.

**Table 3:** Treadmill training speeds and self-selected walking speeds of fast and slow training groups

		**Treadmill training speed (m/s)**					**Final overground SSWS (m/s)**
			
**Group**	**Initial** **overground** **SSWS (m/s)**	**Week 1**	**Week 2**	**Week 3**	**Week 4**	**Mean**	
**Fast**	0.6 ± 0.2 (0.2–1.0)	0.8 ± 0.1 (0.5–1.1)	0.9 ± 0.1 (0.7–1.2)	0.94 ± 0.0 (0.7–1.2)	1.0 ± 0.1 (0.8–1.3)	0.9 ± 0.2 (0.7–1.2)	0.7 ± 0.4 (0.3–1.4)

**Robot**	0.6 ± 0.3 (0.3–1.0)	0.9 ± 0.1 (0.8–1.1)	1.0 ± 0.1 (0.9–1.2)	1.0 ± 0.1 (0.9–1.2)	1.1 ± 0.2 (0.9–1.3)	1.0 ± 0.1 (0.9 ± 1.2)	0.7 ± 0.5 (0.4–1.4)

**Manual**	0.6 ± 0.3 (0.2–0.9)	0.7 ± 0.2 (0.5–0.8	0.8 ± 0.1 (0.7–0.9)	0.9 ± 0.11 (0.7–1.0)	1.0 ± 0.1 (0.8–1.1)	0.8 ± 0.1 (0.7 ± 0.9)	0.7 ± 0.3 (0.3–0.9)

**Slow**	0.6 ± 0.3 (0.3–0.9)	0.5 ± 0.1 (0.3–0.7)	0.6 ± 0.1 (0.3–0.7)	0.6 ± 0.1 (0.3–0.7)	0.6 ± 0.1 (0.4–0.7)	0.6 ± 0.1 (0.3–0.7)	0.7 ± 0.3 (0.3–1.0)

**Robot**	0.7 ± 0.3 (0.3–0.9)	0.6 ± 0.1 (0.6–0.7)	0.7 ± 0.1 (0.6–0.7)	0.7 ± 0.0 (0.6–0.7)	0.7 ± 0.0 (0.7–0.7)	0.7 ± 0.0 (0.7 ± 0.7)	0.8 ± 0.3 (0.3–1.0)

**Manual**	0.6 ± 0.3 (0.3–0.9)	0.5 ± 0.1 (0.3–0.6)	0.5 ± 0.1 (0.3–0.6)	0.5 ± 0.2 (0.3–0.7)	0.6 ± 0.1 (0.4–0.7)	0.5 ± 0.1 (0.3 ± 0.6)	0.7 ± 0.4 (0.3–1.0)

Note: values are mean ± SD (range)

Abbreviations: SSWS = self selected walking speed

## Discussion

The primary purpose of this pilot study was to compare the efficacy of locomotor training implemented using a Lokomat robotic gait orthosis versus manual-BWSTT in a sample with chronic locomotor deficits post-stroke. In conducting this study, we sought to determine the key parameters and reveal challenges in future randomized controlled trials with larger cohorts.

Although statistically significant differences were not apparent between Lokomat and manual groups in this small, pilot trial, our data revealed significantly greater training-related improvements within the Lokomat, but not the manual group. Differential treatment effects produced include: 1) Lokomat group improvements in: self-selected overground walking speed, gait symmetry (SLR_abs_), fast overground walking speed, lower extremity motor impairment (Fugl-Meyer), function (short physical performance battery), and balance (Berg Balance Scale), and 2) manual group improvements solely in balance outcomes (Berg Balance Scale).

### Changes in self-selected walking speed

Modest improvements in self-selected overground walking speed were not unexpected considering that participants were in the chronic post-stroke phase in which recovery is expected to be minimal. The minimal detectable change (MDC) necessary to conclude clinically significant change in gait speed has occurred ranges from 0.07–0.36 m/s in a post-stroke population [39]. Therefore, the 0.1 m/s increase from the mean baseline value revealed in the Lokomat group was not only statistically significant, but also clinically important with an effect size of 0.32. This modest, but significant, effect is especially notable considering the small treatment dose in this preliminary work. Despite the statistically non-significant between-group difference, it is also notable that participants in the Lokomat group increased overground gait speed by 16% over baseline, whereas those in the manual group advanced by only 4.8%. The magnitude of this difference suggests a potential clinical advantage of Lokomat training.

While the overall outcome of this pilot study provides further evidence for the efficacy of locomotor training, the lack of statistical evidence supporting superiority of either Lokomat or manual form of locomotor training highlights inconsistencies between previous studies. Our results agree with investigations during the acute and subacute post-stroke stages using the Lokomat [18] and a robotic gait trainer [17] in which differences were noted within groups, but no differences were identified in the extent of improvement between robot and manual groups. However, in their recent publication, Hornby et al. [20] studied a sample of hemiparetic individuals of greater chronicity (i.e. 4–6 yrs post-stoke) and with lower baseline function (i.e. 0.4 m/s preferred gait speed) than our participant pool and reported greater increases in overground gait speed in a manual-BWSTT group compared with a Lokomat trained group. While speculative at this point, a secondary reduction in cardiorespiratory capacity of chronic stroke survivors [38] suggests that participants with long-term functional deficits may benefit from the aerobic training induced by the higher metabolic cost required for manual-BWSTT [40]. Results of the 6-minute walk test highlight this potential effect. A statistically significant improvement of 34 m, indicative of an aerobic conditioning effect, was found in the manually-trained group of Hornby et al., while a statistically non-significant reduction of 24 m was found in our manually-trained group. Further, a lack of correlation between change scores on the 6 minute walk test and self-selected walking speed suggests that increases in gait speed revealed in the present study are more likely to have resulted from factors other than increased physical capacity, including enhanced neural control, and reflect a change in the underlying locomotor pattern. Moreover, our decision to remove the AFO from all participants during locomotor training appears to have proven effective in improving gait symmetry. In contrast, Hornby and co-workers elected not to focus on improving kinematics, performed locomotor training with AFOs in place, and failed to detect changes in gait symmetry. Future investigations comparing Lokomat versus manual training with a common goal of improved kinematics at different stages of chronicity, may provide more definitive insight into an approximate timeline of beneficial use of one approach over the other.

### Changes in gait symmetry

Our intent throughout locomotor training, whether delivered using the Lokomat or manually, was to normalize gait kinematics during stepping, while simultaneously controlling for variables of BWS and stiffness and treadmill speed. Consistency of training variables in both groups enabled us to discern important differences between motor learning induced by Lokomat and manual-BWSTT. Kinematic improvements in paretic step length symmetry were noted only in the Lokomat group, suggesting greater benefits of consistent, normalized kinesthetic input delivered automatically at a constant guidance force to both lower extremities during gait. In contrast, the inconsistency in both kinematic stepping patterns and manual cues to the hemiparetic leg with therapist-determined level of assistance appears to be a limitation to improvements in gait symmetry, thereby supporting previous research [20].

Further improvements in gait symmetry within the Lokomat group may have arisen from the safe removal of the foot straps in 3 participants. Foot straps are included as part of the standard Lokomat package and are meant to passively set the ankle in neutral and enable foot clearance. Generation of paretic leg propulsive forces is correlated with gait speed, effective step length symmetry [26] and plantarflexion activity during late stance [41]. For these reasons, we strongly encouraged active plantarflexion/push-off and provided verbal and tactile cues in an effort to induce motor learning and voluntary execution of plantarflexion. Examining individual subject changes in gait speed and symmetry, we noted that two of the three participants who were able to train without foot straps demonstrated the most remarkable improvements in step length symmetry. Though we searched for other commonalities between these participants, including sensorimotor impairment scores, initial functional level, location and type of lesion, time since stroke, and age, the one similarity was the removal of the foot strap during training. From a biomechanical perspective, the automated symmetrical step length of the Lokomat would have forced the propulsive forces of the ankle plantarflexors to be initiated posterior to the subject's center of mass (COM) at preswing. A strong relationship between step length symmetry and propulsive force symmetry in addition to the importance of the plantarflexors to propulsive force supports this premise [26,42]. However, further investigations conducted without the use of the foot straps in a larger cohort are necessary to address this issue definitively. At this point we are only able to speculate that a more significant training effect was induced by the opportunity to experience active ankle movement and a normal range of ankle motion while in the Lokomat.

### Changes in balance

Results also revealed significantly improved balance scores producing small to moderate effect sizes on the BBS in both groups. Scores fell within the range in which each 1-pt increase translates to a 6–8% decrease in fall risk. Therefore, the 1.4-pt improvement following Lokomat training and the 4-pt improvement following manual training equates to an 8–14% and 24–32% reduction in fall risk, respectively [43]. These results are not surprising given that treadmill training with or without the Lokomat exposes the central nervous system to several sources of conflicting sensory information, thereby constantly challenging sensory re-weighting processes. Throughout training, proprioceptive inputs from the lower extremity mimic an appropriate stepping pattern on a moving support surface while vestibular and visual cues remain relatively stable. Sensory integration training in such challenging situations may have also translated to improved balance scores in our subject sample. Moreover, the importance of active lateral stabilization to the control of static and dynamic posture and prevention of falls is well established. In this respect, the manual group had a particular advantage in inducing balance improvements with increased lateral freedom compared to the constraints imposed by the Lokomat.

### Comparison of gait training speeds

The second purpose of this study was to assess effects of training at speeds comparable to preferred walking speeds of non-disabled individuals versus speeds comparable to persons post-stroke. Against our hypothesis, our data revealed no differences attributable to training speed on primary or secondary variables. Our hypothesis was based, in part, on a related study by Sullivan et al. [8], who found that training at speeds approaching normal walking speed (0.89 m/s) improved preferred overground gait speed compared with a considerably slower training speed (0.22 m/s). It is possible that since the mean training speed in our slow group at 0.58 m/s was higher, yet more functional, than the slow group in Sullivan et al., the difference between fast and slow groups was not sufficient to reveal training-related differences. Nevertheless, the slow speed in the current study was representative of the pre-intervention comfortable over ground walking speed of the participants, while the fast speed corresponded to a normal (e.g. non-disabled) gait speed of 1.3 m/s [44]. In this view, the two studies are complementary with results supporting training at or above participants' comfortable over ground walking speed rather than non-functional speeds that are below mean overground gait speed of individuals with stroke.

### Study limitations and implications

It may be argued that the automaticity of the Lokomat may have afforded the opportunity to take more steps and, in turn, receive quantitatively more gait training. However, since the mean training speed did not differ between groups, the difference in the number of steps taken would likely be minimal. Moreover, our goal was to evaluate the effectiveness of the Lokomat compared to manual training within a 30 min time frame typically allotted in clinical settings.

As with most pilot studies, the small sample size and resultant low statistical power limit interpretation of this study. However, given that participants demonstrated significant improvements after only 12 treatment sessions in the chronic post-stroke stage, the small to moderate effect sizes are promising. Our results support the original intent of the present pilot study, which was to determine if a larger clinical trial was plausible and should be conducted. This study was different from previous studies because we controlled for many factors such as handrail use, orthotic use, the amount of body weight support, and we placed emphasis on normalizing kinematics during training in order to isolate the specific effects of automated vs. manually-assisted treadmill training. Thus, we were able to show that subjects benefited from training with the Lokomat for a number of performance metrics. One product of our pilot study is tangible results from which to project requisite sample size(s) for future randomized controlled trials designed to definitively evaluate the efficacy of Lokomat compared to manual training. Our primary outcome, self-selected overground walking speed, revealed a between group effect size of 0.59 favoring Lokomat vs. manual training with a probability of 0.6. From this we determined that 51 subjects per group are necessary to detect significant between-group differences. For paretic step length ratio, the demonstrated between-group effect size was 0.73 favoring Lokomat vs. manual training with a probability of 0.70 which translates to a projected sample size of 34 subjects per group. Finally, for fast walking speed, our data revealed a between-group effect size of 0.70 favoring Lokomat vs. manual training at a probability of 0.69 which projects to a sample size of 37 subjects per group to detect between group differences. All sample sizes were projected assuming 80% power at a 5% level of significance.

### Recommendations

While these early, positive findings are encouraging, taken together with the disparate findings reported in the current literature [18-20,45,46], there is a clear need to pursue both the questions regarding efficacy of locomotor training, in general, and robotic-driven locomotor training specifically. We recommend a follow-up study based on our sample size calculations to: probe whether these findings will be reproduced in a larger sample, determine additional differential effects that may not have been revealed in this short pilot trial and test for retention of training effects over an extended period of weeks to months post-training. Further, the advantages and disadvantages of each approach to locomotor training should be weighed in terms of cost-effectiveness, ease of application, and consistency of treatment before definitive conclusions regarding Lokomat use in stroke rehabilitation settings may be drawn. Early evidence favoring locomotor training [9] as an effective intervention post-stroke is tempered by the personnel costs involved (2–4 therapists/trainers), which are unrealistic for the majority of clinical settings. Indeed, many clinics and laboratories that deliver locomotor training depend on considerable volunteer and/or student manpower to simply conduct locomotor training [47] let alone achieve financial feasibility [48]. Equally important is the considerable risk of injury to the trainers representing a significant liability for health care administrators. In this light, equivalent functional outcomes achieved between Lokomat and manual locomotor training represent an favorable result in which Lokomat training may be used in place of manual training to benefit a larger proportion of affected individuals. Further, as demonstrated in the present study, when administered carefully and systematically, robotic-driven motor learning appears to promote adaptation at the level of the locomotor pattern rather than simply offering aerobic conditioning or non-specific changes that contribute to increased gait speed. Long-term retention of these locomotor adaptations is desired and the target of future investigation beyond this initial pilot study. Further research is required to identify the ideal population (i.e. hemiparetic chronicity, severity) for locomotor training, especially robotic-driven approaches to locomotor training, and to elaborate the critical parameters of effective locomotor training, including the ideal amount of variability in kinematic guidance and the most effective schedule for adjusting and ultimately withdrawing kinematic guidance.

## Conclusion

While this pilot study revealed no between-group differences in efficacy of Lokomat versus manual locomotor training, significant within-group effects reveal positive effects of locomotor training and suggest that Lokomat training may offer a potential advantage of this mode over manual BWSTT. A modest dose of Lokomat training is effective for improving overground walking speed and gait symmetry, and other lower extremity impairments and physical function in persons with chronic hemiparesis post-stroke. Consequently, larger, randomized controlled trials are warranted.

## Competing interests

The authors declare that they have no competing interests.

## Authors' contributions

KPW assisted in experimental design, conducted the experiments and data collection, analyzed the data, and was responsible for the initial drafting of the manuscript. CP conceived the study and experimental design, assisted with the experiments and data collection, and helped draft the manuscript. Both authors read and approved the final manuscript.
